# Graefenberg: Or a True Report of the Water-Cure, with an Account of Its Antiquity

**Published:** 1844-10-01

**Authors:** 


### Graefenberg: or, a true Report of the Water-cure, with an Account of its Antiquity. By *Robert Hay Graham*, M.D. Octavo, pp, 232. Longman & Co. 1844.

Truth, we are told, lies in a well—but from the wells of Graefenberg some "mighty big lies," as O'Connell would say, have been dragged up, and scattered over the world! At length, however, we have an approach to truth—or, at least, to candour; for it is clear that Dr. Graham does not consider Priessnitz a god—nor the Water-cure infallible. On the contrary, he has often shewn up the ignorance of the Graefenberg Apollo, and the murderous effects of hydropathy, when unskilfully administered. Still, our author has a monstrous deal more confidence in the "water-cure" than we have—or, indeed, than he is authorized to entertain from the facts which he himself has put on record. The old excuse, however, is always at hand—that we must not confound the *uses* with the *abuses* of a remedy; and this argument is often had recourse to by Dr. Graham himself. The present publication, nevertheless, is decidedly worth all the others put together, not only as to candour, but as to knowledge and learning. We shall therefore take much more notice of it than we have thought it necessary to do of the swarm of hydropathic pamphlets and advertisements that preceded.

Dr. Graham arrived at Graefenberg on the 18th October, 1842, and was immediately introduced to the presiding divinity of the place, whose portrait has been frequently drawn, and need not now be re-painted. After being billetted in one of the huts, the Doctor was visited by Priessnitz—ordered to strip—and enveloped in a wet sheet, without being asked a single question! Dr. G. was surprised to find that two-thirds of the disciples of Priessnitz were young men, the majority of whom were labouring under secondary syphilis! The remainder were chiefly dyspeptics and hypochondriacs.

"Consultations generally take place at table immediately after dinner, when this 'Physician of Nature' is approached with the greatest deference and reverential awe. By some he is looked up to as a demi-god, and not unfrequently so designated; by others, who have not received any relief from his treatment, he is considered as a successful impostor." 15.

Contrary to the practice of some regular physicians who prescribe the same forms of remedy for all diseases, however dissimilar, Priessnitz varies the "WATER-CURE" in almost every individual case, however similar may be the malady.

"He considers the skin to be the principal outlet by which the 'bad stuff,' as he terms it, constituting the disease, is to be expelled. Therefore, when the skin is harsh and dry, and the pores closed, this 'bad stuff' cannot escape. The frequent application of cold water draws it to the surface, opens the pores, and thus facilitates the object. In other words, cold water, by the stimulus of reaction, causes a determination of blood to the skin, and thus becomes a derivative; by which means it greatly increases the functions of this important organ, and solicits the escape of any critical discharge which may take place. He seems not to have any notion, at least he does not admit it, that the application of cold water to the surface ever gives rise to congestion in the large vessels. He says it is warm water which produces this effect, and repels the 'bad stuff;' yet he admits the necessity of feeling warm after the application of cold." 17.

The tactics of Graefenberg are not unworthy of a remark. The regular physician and the barrister are *obliged* to undertake the management of all cases, good or bad. Not so Priessnitz. He makes his selection, rejecting about one-fifth, on an average, of the sick pilgrims—namely, those who are unlikely to be cured, or likely to die! The ADMISSION, therefore, implies a kind of promise of recovery, and inspires hope; while rejection is considered as sentence of death—and doubtless leads occasionally to that event. But Priessnitz has another string to his bow. Every failure on the mountain is ascribed to want of punctuality in obedience to the demi-god's instructions.

When boils recur without relief, it is attributed to "bad blood" as well as "bad stuff." The one preventing the other from getting out properly. Boils come forth in about one-third of the cases, and when recovery follows, it is, of course, from the escape of the "bad stuff" by these safety-valves. He prescribes plenty of food, but no other drink than water. Not even tea is allowed. M. Priessnitz says truly, that his "water-cure" requires "a great deal of strength to enable the patient to go through it."

"How can it be otherwise when carried to the dangerous extreme it is at Graefenberg? This is tacitly implied also by the more experienced of the patients, who say that it is quite sufficient after the first month to do only half what Priessnitz requires; whilst, on the other hand, a relaxed observance of his orders furnishes a plausible excuse for any mishap that may occur." 24.

A lion having been erected on the side of Graefenberg hill, a wag was asked what it meant. He replied that there ought to have been a hog on one side, and a bull on the other, of the statue.

"It would then have signified, that to go through the 'water-cure' at Graefenberg requires the courage of the lion, the strength of the bull, and the stomach of the hog. In every instance of death, which was brought under my notice, I ascertained that it proceeded from congestion, and not from disease—a sufficient proof that the treatment is sometimes carried beyond the endurance of life. This I assert fearless of contradiction, and the most enthusiastic admirers of Priessnitz cannot disprove the fact, however much they may attempt to disguise it." 35.

Dr. Graham considers that the water-cure admits of great modification, as well as combination with proper medicines. The Apollo of the place frankly tells his patients that the "cure" is long—extending to three or four years. "Many of the patients leave in the second year, believing themselves better—others, who have derived no benefit, leave also, endurance being quite exhausted. In these cases the want of success is, of course, attributed to want of patience."

The food at Graefenberg is of the worst and coarsest quality, "such as would be scarcely tolerated in our workhouses." Sour bread, cow-beef without a particle of fat, served up with salted cucumbers or sour-crout, shapeless dumplings "made of the scraps of bread which have been left at table, and soaked in the skimmings of the pot-liquor, sauce made of Dutch herrings, &c.—these are the delicacies of the Graefenberg TABLE D'HÔTE! On Sundays, however, besides a dance, the patients are treated with "baked geese, lean, hard and tough." "They looked miserable, dirty, wet and cold, half-starved in an adjacent pond, *frequently sparing the cook the trouble of killing them.*" From such coarse fare and cold water the Silesian Peasant has realized a fortune (at the age of 42) of *one hundred thousand pounds!* Let the Coopers, the Halfords, the Brodies, and the Chambers, hide their diminished heads!

There is no record kept at Graefenberg of the cures, failures, or deaths. "Col. B., who paid some attention to this matter, and watched the departures in the Autumn of 1842, assured me that the numbers cured fell vastly short of the report." Dr. Graham himself calculated that about one in twenty of the patients were cured. We shall now glance at a few cases.

Miss S. S——, aged 18, fair and most beautiful, in excellent health, and rather plump, accompanied her parents to Graefenberg "on a trip of pleasure." Having caught some of the enthusiasm of the place, she determined to make well better, and took to the water-cure gently at first! For a time, the cold bathing, the mountain air, and other auxiliaries, appeared very pleasant to the neophyte, and she now went the whole hog. Her parents left her at Graefenberg, to get rid of the "bad stuff" in her blood, and returned to England! In the course of a month, the scene began to change. Feverish excitement set in—the glands of the neck swelled—and boils made their appearance. These symptoms were hailed as the harbingers of the crisis, and the expulsion of the "bad stuff." The sweating process was therefore put in practice. Passing over various details, we shall quote the finale, which our readers will do well to shew to their patients who contemplate a pilgrimage to Graefenberg.

"Notwithstanding matters were in this state for some time, Priessnitz expressed himself confident of ultimate success, and said that all was going on most favourably. Seven weeks previous to the fatal termination of the 'cure,' Miss S. S. was removed by her friends to the neighbouring town of Freiwaldau. In seven days after the removal a fever supervened, accompanied with delirium, which lasted for a fortnight. For this two moist sheets were ordered, in immediate succession; the first for half an hour, replaced by a second, in which she remained a full hour. On being taken from the sheet, she was placed in the half or demi-bath, at the low temperature of 50° Fahrenheit. For the first three days after the attack of fever she was well rubbed in the bath with cold water *for two hours;* afterwards for one hour, which treatment was repeated twice in the day. *Whilst in the bath, as well as in the moist sheet, she again complained of pains in the stomach.* The evacuations from the commencement of the fever were red as blood, and continued so to be until death; and latterly, they were nothing but blood. Previously to the fever there was a boil, or 'crisis,' as it was called, on her left breast; it did not suppurate, but receded during the fever. After the fever had left, a vesicular eruption broke out all over the body, but disappeared within a couple of days. At this time, large boils made their appearance, first on the soles of the feet, then on the palms of the hands, afterwards on various parts, or rather all over the body—on the arms, legs, stomach, and sacrum. Even at this period, a fortnight before death, Priessnitz pronounced these boils to be a salutary 'crisis,' and, in spite of all the alarming symptoms, declared that in six weeks she would be perfectly well, and fit to undertake the journey to England. Having been seized with a violent shivering and cramp in her stomach whilst under friction in the half-bath, she insisted on being taken out, at which Priessnitz, when informed of it, became very angry, and the next day sent one of his own women with strict orders to prosecute this operation until she became warm.

"The moist sheet and the half-bath were persevered with twice a day until within two days of her decease. *During the last three days she vomited blood.* No other remedies were employed to relieve the patient, and none to sustain life. On the night of the second day after the discontinuance of the treatment, this hapless young lady expired in the arms of her attendant, whilst being raised in bed, the blood at the same time gushing out of her mouth and nostrils." 53.

We do not quote this melancholy case to shew the fatal effects which sometimes result from the COLD-WATER cure, but to prove the total ignorance of Priessnitz, and the absurdity of believing one word that he says, or relying on one atom of his prognostic or diagnostic skill. On opening the body of this murdered lady, the viscera were all found to be sound—no enlargement of the mesenteric glands. The stomach was found to be coated with a thick brownish mass, extending into the jejunum, and the vessels throughout the alimentary canal "were in a high state of congestion."

"At a subsequent period, when my friend Captain Wolff informed Priessnitz, that it was decidedly my opinion she died from congestion, induced by the treatment, especially the moist sheet, he shrugged his shoulders, and replied, that 'something gave way in her inside, which caused death. That it was his practice to judge of the inside by the skin, but that he was restricted in his observations in her case, and therefore could not tell what was going on withinside." 55.

We commiserate the feelings of the wretched parents who—

"Left in that fatal lair the tender fawn,"

to die among strangers in a foreign land, consigned to an early tomb by the murderous hand of an ignorant butcher, who "mimicked the tone of her voice, and her retiring modesty, when he once attempted to remove her bathing dress."

"He afterwards ridiculed the English ladies for using bathing dresses at all, so different from the custom of his own countrywomen. And all this was said and done with a sort of acting or imitating their manners, highly amusing to his hearers, who burst out into repeated shouts of laughter. Such is the great, the immortal Priessnitz! *Proh pudor!*" 55.

Such is the school to which our fair countrywomen resort to foster their morals and recover their health!

*Case the second.*—The Princess L——, affected with fluor albus, which Priessnitz pronounced to be cancer of the womb!! After going through the processes of the water-cure, the Princess recovered perfectly from CANCER UTERI! She afterwards became pregnant, and, at the period of her accouchement, "the hip-bath was prescribed to arrest the false pains and accelerate the labour." In the course of two hours, labour-pains came on, and she was safely delivered. The hip-bath was ordered the following day "to draw down the bad stuff," and strengthen the parts. The wet bandage was also applied to the abdomen. Whilst in the hip-bath, the Princess complained of pains in the region of the uterus, and re-action did not take place. The third day, the hip-bath repeated for a longer period. The pain became more violent, inflammation more intense, the lacteal secretion was suppressed, and, in 24 hours she was a corpse. The Prince left Graefenberg, bewailing his loss, and openly accusing Priessnitz of destroying the life of his consort. Apollo excused himself by alleging that his orders could not have been strictly obeyed!

*Case* 3.—A gentleman of fortune was attacked with diarrhœa, while at Graefenberg. To promote the descent of the "bad stuff," he was ordered the hip-bath, and to repeat it every two hours. Evacuations of blood succeeded, which were pronounced to be favourable symptoms. In about a week he died!

*Case* 4.—A Prussian Captain had been afflicted with asthma for several years. He took the wet sheet, in which he perspired, and on going into the cold bath, immediately died!

*Case* 5.—M. Dzubo, a Captain in the Austrian service, had been some months under treatment, when the crisis appeared in the form of a tumor in the throat. It burst internally and he instantly expired. There is no surgery employed in Graefenberg. Nature and cold water are to do all. The lancet would probably have saved the Captain's life.

"Two-thirds of his patients are said to be syphilitic. Of these, almost every one has been over-dosed with mercury, and the majority of them suffer solely from that form of the disease, which, when the virus is destroyed, may be called mercurio-syphilitic, being the effects of mercury. Under the 'water-treatment,' as practised at Graefenberg, it requires from six months to two or three years to cure these secondary symptoms. No doubt the progress of the cure is retarded by the quality of the food, and the too long and too frequent application of cold." 59.

*Case* 6.—Capt. Wolff, a great friend of Dr. Graham's, and under the water-cure with him, went to Graefenberg for the treatment of an affection of the head, consisting of heat at the vertex—cephalalgia—muscæ volitantes, dimness of vision, constipation, inflamed face and nose, &c. under which he had laboured for several years. He had been at Graefenberg nine months, and left at the same time as Dr. Graham did. "He went through the entire course, with occasional variations." He was an enthusiastic admirer of Priessnitz, and had unbounded confidence in the water-cure.

"One day, entering my apartment radiant with joy, he announced that the 'bad stuff' was at last 'drawn down,' for that he had evacuated a large quantity of slime mixed with cheese-like matter. I could not even then convince him, that this was the effect of the cold hip-bath repelling the blood towards the intestines, and thus causing congestion, and increasing the malady." 67.

The Captain pursued the directions given him, rising at three o'clock in Winter mornings, to be enveloped in the wet sheet, and taking the cold plunging-bath immediately afterwards!!

"Since my return to England I have received a letter containing the following remarks:—

'. . . . What further effects has the 'water-cure' produced on your constitution? As for me, alas, it has not yet obtained the wished-for success. I am almost always in the same condition, suffering still from dyspepsia, the 'mouches volantes,' and everything else, the same as before. You were indeed a true, but, to me, ill-omened prophet!" 67.

We shall now take a glance at Dr. Graham's own history.

*Case* 7.—Dr. G. aged 52, arrived at Graefenberg, as was before stated, on the 18th of October, 1842. He had been subject to gout, at intervals, for ten years previously—the fits influenced by moral as well as physical causes. There was a dash of rheumatism in the gouty diathesis. Although he had a good appetite, and was inclined to corpulence, he felt weak, with somewhat dry and harsh skin, cramps and bad nights. The pulse was contracted, varying from 70 to 80, with palpitation, dyspnœa, perspiration and exhaustion on taking exercise. The weather, at Graefenberg, was then keen and frosty, the mountains covered with snow.

"At five in the morning I was enveloped in the moist sheet. The sensation of cold was at first so intense, that it seemed as if I was packed in ice and snow. A quivering of the muscles and chattering of the teeth came on, similar to that which is experienced in a fit of the ague. In two or three minutes, this sensation passed away, the heat of the body having gradually created a sort of vapour-bath, when I was somewhat reconciled to my new situation. My pulse was rapidly brought down to 60, and did not afterwards rise above 66. In three quarters of an hour, the bath-attendant, Frantz, came and found me in a slight perspiration, when I got up and was washed for two or three minutes in the half-bath, the extreme chill of the water having been taken off so as to bring it to about 50° or 55° Fahrenheit. At eleven I was rubbed down with the wet sheet, which was succeeded by a foot-bath for ten or fifteen minutes, of extremely cold water, about two inches in depth. The effect of the cold on the soles of the feet was such that I could scarcely endure it, and the feet became of a bright crimson hue. At five in the afternoon the treatment was repeated, the moist sheet for an hour, and then the half-bath.

"Next day, the hip-bath was substituted for the foot-bath. This was also intensely cold, causing a stinging, tingling sensation, as if the points of innumerable fine needles were everywhere penetrating the skin as it came into contact with the water. The same bright crimson colour appeared as before. This sensation, together with the look of the skin, were deemed highly propitious. Such was the course of treatment I underwent, without much deviation, during the first week. I could not become reconciled to the moist sheet; it frequently brought down my pulse to 50; hence, I sometimes shirked it in the afternoon, and used instead the hip-bath, and friction with the dripping sheet. I had a great inclination for a sweat in the blanket." 70.

We cannot pursue the details of the treatment which our author underwent, till one day (Nov. 4) he stumbled over the edge of the plunging-bath, and received a contusion in the left thigh, from which he went through the miseries of the damned till a severe attack of gout came on.

"On the following day, Sunday, November 27, the gout fully declared itself. The wet bandage had caused a vesicular eruption and inflammation of the skin, and, without doubt, brought on the disease, which now proceeded rapidly, and with excruciating pain. I felt as if my bones were being crushed and ground between two mill-stones. I did not, however, as on former occasions, experience that burning heat in the articulation of the great toe, as though a drop of molten lead were boiling and hissing in the joint, or as if it were being rended and riven asunder with wedges of red-hot iron. The burning heat was located in, or drawn to the skin. The left knee was also attacked, and became enlarged, very painful, and not admitting of the slightest motion. I also suffered much pain in the loins, especially about the left kidney. In this state, too intently occupied with my sufferings, sleep was banished from my eyes. My kind friend proposed to sit up with me, which I could not permit. The night was, therefore passed in solitude, and its stillness only broken by my half-suppressed sighs and groans." 75.

We will not fatigue the reader with the plans adopted by Priessnitz to cure Dr. G. of the gout—or of the religious obedience which the learned Doctor paid to the dictates of the ignorant Charlatan. In the course of the cure we find the following note.

"I was now sadly reduced by the disease, by the severity of the treatment, and by my great abstinence,—my food consisting merely of a small quantity of bread and milk, and, at dinner, a sort of hasty pudding. The frequent application of the moist sheet, during the first attack, had reduced my pulse, whilst I was in it, generally to 53, sometimes to 48. I had a short, dry, hard cough; sneezing, and a constant singing in my ears, resembling the chirping of birds, which last effect I have not even yet entirely got rid of. Under these circumstances, and as the moist sheet was not imperative, I resolved to abstain from its further use. In vain my friend, Capt. Wolff, urged me to continue it; my reply was, that if I did, he would have to lay me by the side of poor Miss S. S.; therefore, instead of the moist sheet, I was enveloped every day, from six to seven or eight hours, in the blanket, and then rubbed for half an hour in the bath, at 55° or 60° Fahrenheit." 78.

Scarcely convalescing from a third attack of gout, our worthy Doctor determined to make his escape from Graefenberg, though in the very depth of winter. Bandaged in wet clothes he reached Breslau, and at last Dresden, where he had left his son at school. The following passage speaks for itself.

"Worn out with sickness and fatigue, I returned to the hotel which I had put up at previously to my journey to Graefenberg. As the bandages had not been renewed since I left Breslau, they had become perfectly dry, and my legs were thickly covered with a silvery scurf. Having bathed them, and replaced the bandages, I sent for my son, a boy eleven years old, whom I had placed here at school. On entering the room, and approaching toward me, he all at once stopped short, gazed in mute astonishment, and hesitated whether to advance or not; and, even after I had embraced him, his steadfast look was riveted upon me, until at last he burst into tears. Affected at this conduct of the child, my first impression was that he was unhappy at school, which drew from me the exclamation, that if such were the case he should return with me to England. 'Why papa,' he at length sobbed out, 'you look so thin that I did not know you—I could not believe it was you.' I relate this circumstance merely to show how altered I must have been in that short space of time, for my son not to have recognised me. He was not singular, however, in this respect, for it was the case with every one, even the domestics at the hotel. The change having taken place gradually, I was familiarized with it, and quite unconscious of the extent to which it had gone." 81.

The Doctor reached his native home; but had not been there more than a few days, "when gout came on in both his feet and knees." "Thus I had four or rather five distinct attacks of gout in little more than three months, in spite of Priessnitz's prediction that, after the second fit, *I should never have it again.*"

The greater part of the volume before us is occupied with reflections on the nature and treatment of gout—most of them sensible and judicious—and with researches on the "WATER-CURE," as set forth in the writings of Drs. Hahn, Floyer, Baynard, Ellison, Vander Heyden, J. King, Rush, Kinglake, Smith, Todaro, &c. &c. which will be read with some curiosity by the public at large, as well as by the profession; but we have confined our analysis to the "Personal Narrative" of our author, as more likely to prove beneficial to the reader than speculations or researches. If this narrative were converted into a homily, and read in the churches of the United Kingdom every Sunday, a *few* might be deterred by their *fears* from making a pilgrimage to Silesia; but when we see grave, learned, and even experienced physicians and surgeons discharge from their brains every particle of the reason which God gave them, and the knowledge which they had acquired from books and bedside, to run to Graefenberg and risk their lives under the experiments of an ignorant peasant, how can we wonder at non-professional individuals swallowing the miracles said to be performed on the Silesian Mountain, and running thither in quest of impossible cures, or for the treatment of maladies that could be more safely cured at home? Every medical man who thus makes an ass of himself, materially injures his profession, and tends, as far as he can, to bring it into contempt with the public! We may pity and despise the tomfooleries of a Burdett, who patronised successively the quackeries of a Morison, a Long, and a Priessnitz, dying at last in the wet harness of Graefenberg;—but we cannot help entertaining some other feelings towards those of our own profession, who deeply injure medical science by prostrating themselves at the shrine of ignorance, impudence, or imposture, in the forms of Hydropathy, Homeopathy, and Mesmerism. We hope the sufferings of Dr. Graham will prove a salutary lesson, not only to himself, but to others, both in and out of the profession. We certainly think that he has had a narrow escape from the fate of the young English lady who perished in a foreign land, under the WATER-DÆMON OF GRAEFENBERG!

